# Allergen-specific immunotherapy and evidence: A European regulatory perspective 

**DOI:** 10.5414/ALX02413E

**Published:** 2023-12-12

**Authors:** Detlef Bartel, Andreas Bonertz, Diana Hartenstein, Stefan Kaul, Iris Lauer, Christina Reeb, Karen Rösner-Friese, Katja Sliva, Julia Zimmer, Stefan Vieths, Vera Mahler

**Affiliations:** Paul-Ehrlich-Institut, Langen, Germany

**Keywords:** SCIT, SLIT, Directive 2001/83/EC, marketing authorization, in-house reference preparation, cross-product comparability, manufacturing process, clinical relevance, Therapy Allergen Ordinance (TAO), European Pharmacopoeia

## Abstract

Allergen immunotherapy (AIT) has been performed for 112 years. In this article we summarize regulatory standards and challenges based on scientific evidence on AIT. Most crucial and timely aspects concerning AIT are addressed from the regulatory perspective of the authors as employees of a national competent authority in Europe: (1) product specificity; (2) clinical efficacy; (3) treatment for adults and children (needs for extrapolation); (4) allergen exposure chambers; (5) biomarkers; (6) standardization; (7) real-world evidence; (8) independent official batch release (benefit and challenges); (9) harmonization on the EU level. The Paul-Ehrlich-Institut (PEI), the Federal Institute for Vaccines and Biomedicines, in Langen near Frankfurt/Main in Germany, examines and evaluates the benefits and risks of AIT products within the course of clinical development, marketing authorization, and subsequently throughout their entire life cycle to ensure high-quality, safe, and effective AIT products.

## Introduction 

Allergen immunotherapy (AIT) has been performed for 112 years. The scientific evidence gained in the course of time since the beginning of AIT is the basis for the regulatory assessment and authorization of allergen products. Allergens have been subject to European law since 1989 (Directive 89/342/EEC) [1]. According to the definition in Directive 2001/83/EC [2], the current Community code relating to medicinal products for human use into which the Directive 89/342/EEC has been incorporated, both test and therapy allergens are medicinal products. The Directive had to be transposed into national legislation in all EU member states (e.g., the German Medicinal Products Act (Arzneimittelgesetz; AMG) [3] in Germany). 

According to Article 6 of this European Directive, a medicinal product may not be placed on the market in a member state unless a marketing authorization (MA) has been granted by the competent authority of that member state. However, the different use of Article 5 (A member state may, in accordance with legislation in force and to fulfil special needs, exclude from the provisions of this Directive medicinal products supplied in response to a bona fide unsolicited order, formulated in accordance with the specifications of an licensed healthcare professional and for use by his or her individual patients on his or her direct personal responsibility.) of the Directive lead to different regulatory approaches to allergen products in different EU member states, which are currently seeking harmonization. 

**Figure 1:**
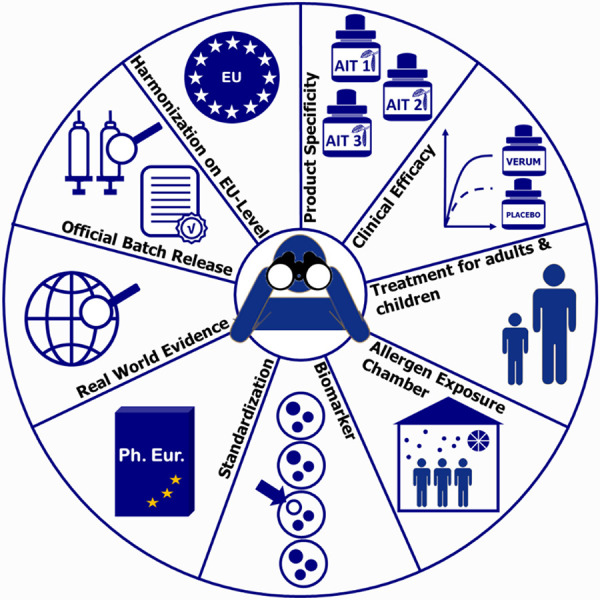
Important topics and current challenges in allergen-specific immunotherapy from the regulator´s perspective.

In this article we summarize regulatory standards and challenges based on scientific evidence on AIT (Figure 1). 

## Product specificity 

Different AIT products containing intact allergens or chemically modified allergens (allergoids) as active components are commercially available for subcutaneous, sublingual and oral application in Europe. Allergen preparations used for subcutaneous immunotherapy (SCIT) are either allergoids or non-modified (“native”) extracts which are frequently physically coupled whereas for sublingual immunotherapy (SLIT) mostly “native” extracts with an unmodified allergen conformation are used, as aqueous solutions or tablets [4]. For oral application (oral immunotherapy (OIT)), a defatted peanut powder containing native allergens has been authorized for peanut immunotherapy in the EU and the U.S. [4]. 

Furthermore, novel AIT products are being developed and investigated in clinical trials, e.g., mannan-allergoid conjugated preparations, as well as nanoparticles encapsulating the allergens and virus-like particle (VLP) co-administrating the allergens, which are promising candidates for future MA [5, 6]. 

All these AIT products consist of individually different compositions and formulations, depending on the manufacturing process, possible modifications, and adjuvants used. As allergen products derived from natural sources, such as pollen, insects, mites, or foods, are inherently variable in their qualitative and quantitative composition, and the distinct manufacturing process applied for their production is highly individual, they are not directly comparable and display strongly differing and individual qualitative and quantitative characteristics, even for the same allergen source [7]. Although each product has to be characterized by a product-specific in-house reference preparation (IHRP) with a defined protein concentration and strength based on product-specific human sera-based potency assays [8, 9], a cross-product comparability (CPC) is currently not possible, as different methods (“in-house assays”) and manufacturer-specific units are used [10]. With the determination of individual allergens (usually major allergens) using standardized, validated methods, a limited quantitative comparison between different AIT products regarding these allergens becomes possible for the same allergen source (see chapter standardization below). 

While such information undoubtedly adds important characteristics on the individual AIT product’s composition, additional factors critical for the expected properties of any biological medicinal product with regard to its efficacy and safety will still strongly depend on the individual manufacturing process and the comprehensive characterization of the product regarding its qualitative and quantitative parameters. As a result, clinical efficacy and safety of any individual product cannot be transferred to other AIT products, even if they derive from equal source materials and are presented in equal formulations (except for products fulfilling the criteria of homologous groups as has been specified in [8, 11]). Products are not alike, if they are manufactured according to a different manufacturing process, which will almost exclusively be the case if different manufacturers are involved. 

AIT allergen products contain a mixture of allergenic and non-allergenic components of native or modified allergens in a wide variety of formulations. Consequently, due to the limited cross-product comparability at the qualitative level for the different types of AIT allergen products, the informative value of meta-analyses on allergen sources with regard to the clinical efficacy of different AIT products is also limited. 

## Clinical efficacy 

Product-specific efficacy data are summarized in the product characteristics. Information on administration should result from the clinical development and be described in the summary of product characteristics (SmPC) and package leaflet (PL). A product-specific appraisal of the individual AIT preparations is recommended [12]. 

To provide evidence that AIT is effective, a review of the overall study data is crucial; confirmation of results is required and study data must prove robust. Meta-analyses confirm a generally well-documented efficacy of AIT in allergic rhinitis/rhinoconjunctivitis (AR/ARC) and allergic asthma (AA) [12]. However, in line with regulatory requirements, evidence of efficacy can be demonstrated only on a product-specific basis. This also applies for the efficacy of the various application formulas (e.g., comparison of SLIT versus SCIT); a generalization is not possible. 

A clinical trial for AIT can evaluate either efficacy in AR/ARC or efficacy in AA as the primary objective, not both together [11, 13]. 

For each study, the primary endpoint, including a threshold for the difference in outcomes between active treatment and placebo treatment that is considered clinically relevant, must be pre-specified and justified. According to EMA Guideline CHMP/EWP/18504/2006 [11] the primary endpoint has to reflect effects on clinical symptoms as well as effects on medication use. To date, no validated score that combines symptoms and medications is used. Therefore, suitability of the chosen combined score, including the individual weighting of symptoms versus medications, as well as the time point and duration of the evaluation period, have to be defined in advance in the study protocol. Due to different types of scores used and the fact that some of the scores used do not weigh intake of rescue medication and symptom reduction equally, results of different studies often cannot be compared directly. In order to avoid false conclusions, the selected endpoint needs to be carefully reviewed for each study to understand the clinical impact of the study treatment compared to the placebo effect. 

The evaluation period should cover the period of high allergen exposure (e.g., pollen season for seasonal allergens). Study and treatment duration will affect the approvable indication. 

In clinical trials, AIT is considered an add-on therapy. The comparison of effects gained with the investigational medicinal product (IMP) in comparison to placebo is of major importance, especially because a variable and potentially large placebo effect is often reported in AIT trials [14], and needs to be distinguished from real immunological treatment effects. It is observed that subjects with perennial allergies, e.g., against cats or mites, seem to experience stronger placebo effects compared to subjects treated for seasonal pollen allergies. 

Statistical significance over placebo is a prerequisite for MA. Nevertheless, the threshold of effect above placebo that is considered clinically relevant for the concerned product has to be determined and properly justified. There is no general threshold expressed in percentage which is accepted by European regulatory authorities as a criterion of clinical relevance. For AIT with its potential of systemic allergic adverse reactions, a stronger effect is expected than with symptomatic treatment. However, an effect of 20% over placebo as a general threshold, as proposed by the World Allergy Organization (WAO) taskforce in the past [15] and frequently quoted, is not generally considered meaningful. The translation of a percentage value into an absolute value strongly depends on the absolute reference value achieved by placebo. In fact, a 20% difference with a low placebo effect means only a small absolute treatment effect compared to 20% with a high placebo effect. Therefore, efficacy of the product should always be defined in absolute values compared to placebo. From the clinical trial data it must be clear what the analyzed difference between the effect of the investigational product and placebo means for patient improvement when translated into a clinical effect, to decide on its clinical relevance. For AR/ARC, a minimal difference considered perceivable for a patient with seasonal allergies is a decrease of minimum one severity class (from moderate to mild or from severe to moderate) in two symptoms or of two severity classes (from severe to mild) in one symptom. For house dust mite allergy, which generally presents with less pronounced AR symptoms and is known to be difficult to treat, the German competent authority, the Paul-Ehrlich-Institut (PEI), considers a decrease of minimum one severity class in one symptom, if averaged over 12 months, as a minimal effect. 

It is concluded that due to the fact that clinical trials use different combined symptom-medication scores, generalization is not possible and clinical trial results need to be translated into symptoms or medication effects individually per trial and product. 

To assess clinical efficacy in allergic asthma and food allergy, new endpoints are and will be developed in close exchange between science and regulation. For example, for treatment of allergic asthma, the difference in exacerbations between subjects treated with investigational product compared to those receiving placebo have been analyzed. There are also other endpoints like lung function, composite scores, number of exacerbations or reduced need for controller medication which could be considered as primary endpoints [13]. For food allergy, the gold standard for diagnosis and assessment of efficacy is the double-blind placebo-controlled food challenge (DBPCFC). However, protection from reactions in case of accidental intake, improved quality of life, and sustained efficacy after end of treatment are also relevant clinical efficacy parameters. Regardless of the choice of the primary efficacy parameter, the applicant should provide a definition of a clinically meaningful effect. 

## Treatment for adults and children: Needs for extrapolation 

With regard to the regulatory requirements in the development of medicinal products, children belong to a so-called “special population” for which additional regulations apply. First of all, the Regulation (EC) No. 1901/2006 on medicinal products for pediatric use [16] has to be considered. This regulation was set up to ensure that in the development of medicinal products the special therapeutic needs of children as well as the special vulnerability of this population are considered [17]. One prerequisite for an MA of medicinal products and thus also for products for AIT laid down in this regulation is the need to provide an approved pediatric investigation plan (PIP) within the dossier for application for MA. These PIPs have to be assessed and approved by the pediatric committee (PDCO) of the EMA [18]. With the Therapy Allergen Ordinance (TAO), which came into force in Germany in 2008, all allergen products underlying the TAO needed a PIP for the MA application which had to be submitted until 2010, meaning that a lot of PIPs all regarding allergen products for AIT were submitted to the PDCO and needed an approval on relatively short notice [19]. To simplify the assessment of this “flood” of PIPs, a Standard PIP for AIT products (currently in the 4th Revision) [20] was developed. During this development, the PDCO concluded that for children only the assumed disease-modifying effect is decisive and outweighs the risks associated with AIT. However, up to now, only few data exist for the disease-modifying effect in general and even less in children. In addition, there are no studies allowing for the conclusion that products which proved effective in adults would be similarly effective in children, preventing to this day an extrapolation from adults to children [17]. Thus, there is a high unmet need to extrapolate efficacy data from adults to children. To establish a basis of data to compare efficacy in adults and children, the Standard PIP lays down that every manufacturer of AIT products has to select one product out of their portfolio (the so-called “selected product”) and to perform both one long-term study (i.e., double-blind, placebo-controlled with 3 years of treatment and 2 years of follow-up) in adults and one long-term study in children with this product. Depending on the results of such studies, this data base will then ideally allow extrapolation from adults to children. Therefore, all other products of the manufacturers were granted a deferral according to Articles 20 and 21 of Regulation (EC) No. 1901/2006 until performance of these two studies. After complying this prerequisite of the Standard PIP, the manufacturer can modify the PIPs for all other products, and then all other study designs as outlined by the Guideline on the Clinical Development of Products for Specific Immunotherapy for the Treatment of Allergic Diseases [11] are possible. 

Unfortunately, none of the required long-term studies in children with products subjected to TAO has started yet. To elucidate the reasons and searching for solutions, two meetings were initiated by the PDCO, one workshop on AIT for Children, held at EMA in 2018 and, as a follow-up meeting, a multi-stakeholder Meeting on AIT for Children held at the PEI in 2019 [17]. At this meeting, there was consensus among clinicians and investigators that long-term studies are considered feasible in children for SLIT but not for SCIT products, as the requirement for 3 years of placebo injections was seen as a too high burden. In the end, manufacturers were encouraged to collaborate among themselves to explore new biomarkers, study designs, study networks, and the use of allergen exposure chambers at least in adults. 

Regarding the need for extrapolation, it was concluded that extrapolation is not yet justified, and an approach to extrapolate efficacy data from adults and to perform only safety studies in a pediatric population will only be possible after scientific proof of concept that this extrapolation is feasible. If this proof has been obtained with SLIT products and a long-term efficacy of SLIT products has been convincingly demonstrated in adults and children, long-term efficacy in the pediatric population may be extrapolated from data generated with the respective product in adults. 

## Allergen exposure chambers 

Allergen exposure chambers (AECs) enable a controlled, uniform, and standardized exposure to inhalant allergens in a steady environment with regards to, e.g., temperature, humidity, air pressure, and foreign particles. The AEC model intends to mimic natural exposure to airborne allergens in respiratory allergic diseases like AR, conjunctivitis, and asthma. AECs can be used to investigate treatment effects of antiallergic medication and AIT. AEC technology can overcome drawbacks that are inherent to traditional field studies. Classical field studies utilize everyday life exposure to the offending allergen, but the actual allergen exposure is highly variable due to internal (subject-related) and external factors like geographically and seasonally changing pollen counts and weather. The control of these variables is the hallmark of AEC technology. Furthermore, experimental allergen exposure allows for close subject monitoring, studying disease mechanisms, and investigating dose effects. 

The first AEC facilities date back to the 1980s and were employed to evaluate, e.g., onset, duration of action, and dose effects of symptomatic medication for AR. Since then, the AEC model has been continuously further developed and established for clinical AIT research. To date, facilities are operated in Europe, North America, and Japan. Despite the wide spread, their overall number is still limited and their capacity varies from a few to over 100 patients per chamber, which may be a limiting factor in the scope of clinical studies. 

AEC facilities in operation underwent thorough technical validations to proof uniformity with regards to stable and reproducible allergen exposure and constant environmental conditions. Technical validation protocols are individualized for each AEC center and allergen source and have been developed for perennial (e.g., cat, house dust mite) and various seasonal airborne allergen sources like birch, oak, ragweed, and grass pollen. In addition, center-specific protocols are in place to enable a reliable and reproducible symptom induction. Due to the unique technical and engineering features of each AEC center and the lack of international validation standards, study results gathered in different facilities are not comparable. In addition, transferability of AEC study findings to real-life conditions has not been proven yet. In light of these shortcomings, the AEC model is accepted for phase II trials but not yet for confirmatory (phase III) efficacy trials as laid down by the EMA Guideline on the “Clinical Development of Products for Specific Immunotherapy for the Treatment of Allergic Diseases” [11]. AEC technology was therefore utilized in multiple phase II AIT trials [21, 22]. It was further validated for cat [23, 24] and house dust mite allergen exposure [25, 26], but is still underexploited in the field of AIT product development. The latter prompted a European Academy for Allergy and Clinical Immunology (EAACI) Task Force initiative with the aim to identify and define technical and clinical validation standards. The EAACI Task Force identified three major fields for the validation process [27, 28]:

1. The standardization of the challenge procedures and assessments.
2. The validation of intra-AEC reproducibility and inter-AEC variability and reproducibility.
3. The evaluation of the comparability with field trials.

Point 3 is especially important for a possible acceptance of confirmatory AEC studies within the licensing process, since the main criticism of the chamber setting is the artificial set-up not resembling real-life exposure (e.g., seasonal priming, weather conditions, individual lifestyle). Hybrid trials are needed to shed light on the vital question whether effects measured in the AEC setting correlate with the treatment outcome during everyday exposure. Hybrid trials are designed to cover efficacy evaluation during both natural season and experimental (AEC) exposure for the same patient population. 

The performance of hybrid trials and further validation efforts are highly encouraged since it is certainly recognized that the AEC technology could be a powerful tool in AIT product development. 

## Biomarkers 

Biomarkers (BMs) are objectively detectable biological indicators whose (elevated) presence indicates a disease [29]. The use of BMs is firmly integrated in the regulation of medicinal products through respective guidance [30, 31] and has the potential to facilitate the availability of safer and more effective medicines, to guide dose selection, and to improve their risk-benefit profile. The ideal BM should be easily detectable and reproducible. In the treatment of allergies, two main options are available: In contrast to the conventional symptomatic pharmacotherapy, AIT aims at modifying the disease with the goal of tolerance induction and allergen insensitivity. AIT is currently applied for respiratory, insect venom, and food allergy. However, not all patients respond equally to the therapy. Contributing to the varying response and success for AIT is the complexity of the disease, with partly overlapping allergic disease entities comprising AR, conjunctivitis, chronic rhinosinusitis, allergic asthma, insect venom allergy, food allergy, and atopic dermatitis of varying degrees. Further, the disease might be influenced by endogenous and exogenous cofactors, e.g., patient-specific diet, infections, exposure to antibiotics and chemicals, microbiome composition, and genetic and epigenetic factors influencing multiple molecular pathways [32]. BMs capable of predicting AIT efficacy which enable distinction of responders from non-responders would potentially greatly simplify the verification of efficacy through clinical trials. Availability of reliably predictive BMs would also transform the field from a regulator’s perspective providing a directly measurable variable for monitoring treatment efficacy. Currently, allergy is diagnosed by detailed symptom history and confirmation of relevant IgE sensitization and based on this the indication for AIT is established. Given the complexity of underlying disease parameters, a robust BM may likely be a combination of factors that contribute to antigen-specific tolerance induction and desensitization. Literature review revealed numerous scientific publications demonstrating that the need for BMs has been recognized, but also highlights the problem that due to the heterogeneity of allergic diseases, the numerous BMs identified are mostly not clinically relevant or the clinical relevance for prediction of AIT efficacy has not been validated. 

In 2017, an EAACI Task Force reviewed the existing literature regarding potential candidate BMs and identified a promising list of BMs attributable to seven domains [33]: (i) IgE; (ii) IgG subclasses; (iii) serum inhibitory activity for IgE; (iv) basophil activation; (v) cytokines and chemokines; (vi) cellular markers (T regulatory cells, B regulatory cells, and dendritic cells); and (vii) in vivo BMs. This published list could be supplemented by “OMICS”, which summarizes a group of molecular methods (e.g., transcriptomics or proteomics) aiming at identifying molecular information lying behind pathogenesis. The rapid progress of OMICS technologies is leading to advances in the molecular characterization of the relationships and courses of complex human diseases, including allergies. This new type of precision medicine is being driven by multi-OMICS analyses in genomics, transcriptomics, epigenomics, proteomics, metabolomics, and more. In the context of asthma, breathomics (studying the contents of exhaled breath of an individual) is also being investigated for potential BMs [34]. A recent report describes nasal DNA methylation to have good prediction power for allergic diseases in adolescents [35] and being able to distinguish symptomatic from asymptomatic IgE sensitization. In this study, a multi-OMICS analysis combining genetic, epigenetic, and environmental factors was performed. 

Another interesting and emerging field is the identification of microRNAs (miRNAs) in the context of allergic diseases. miRNA have been proposed as BMs for, e.g., asthma [36] and atopic dermatitis [37], to mention only two. 

Although the results are promising, even in the OMICS-area they are not robust yet. 

From a regulator’s perspective, reliable BMs must be accurately measured by validated methods in accordance with the regulatory guidelines [38] and a clear correlation of BMs with clinical efficacy of AIT must be demonstrated before these BMs can serve as accepted primary endpoints in clinical studies. Despite a number of approaches and advances in precision medicine, there is currently no validated and generally accepted BM candidate that could predict therapeutic success of AIT. 

## Standardization 

In the field of allergology, the term “standardization” is commonly used synonymously for two different challenges: firstly product-specific standardization (PSS), enabling reproducible production of batches of the same allergen product, and secondly cross-product comparability (CPC), enabling comparison of products from different manufacturers [10]. In Europe, PSS has been significantly pushed forward in the last decades by corresponding regulatory requirements. However, a cornerstone of PSS has been and still is comparison of IgE-binding capacity of a new batch to a representative product batch, the in-house reference preparation (IHRP). This combination of product-specific references and human sera-based potency assays has impeded CPC, because only the combination of a common reference standard and a standard method generates comparable results. The recently concluded project BSP090 has now provided these prerequisites for birch pollen and timothy grass pollen-based allergen products by establishment and validation of recombinant reference standards for Bet v 1 and Phl p 5 and corresponding allergen-specific ELISAs [39, 40]. The allergen standards have been available at the EDQM since 2012. The General Text describing the standard methods was implemented in the Ph. Eur. on January 01, 2023 for the Bet v 1-specific assay and will probably be implemented in 2024 for the Phl p 5-specific assay. Notably, Ph. Eur. General Texts are not legally binding per se, but have to be referenced in a Ph. Eur. monograph to become mandatory [41]. Therefore, the General Monograph on Allergen Products is currently in revision to request the use of reference standard and standard assay as soon as the respective material is implemented for a certain allergen. For therapeutic allergen products based on either timothy grass pollen or birch pollen, the revised monograph will request labeling of major allergen content, calculated per dose of the respective maintenance dose of the allergen product. 

In view of the variety of allergen source materials used in therapeutic allergen products, the result of BSP090 can only be regarded as a first, though important step for CPC in Europe. In comparison, CPC in, e.g., the USA covers a much larger range of different allergens, based on standard assays and standard extracts [42]. Hence, the experience collected in the establishment of the first Ph. Eur. allergen standards and allergen-specific standard methods should be utilized to extend CPC in Europe to other important allergens, covering both aeroallergens and food allergens. 

## Real-world evidence 

Patient registries, healthcare databases, electronic health records, and patient-generated data from wearables or apps provide a vast amount of information on patients reflecting so-called real-world data (RWD). 

In the field of allergology, a variety of “hay fever” apps provide users with information such as pollen count forecast and collect data on the users’ symptoms and medication use [43]. These apps can be used by anyone; thus, the collected data are self-reported, meaning that they lack proof of confirmed diagnoses and are without any claim of being exhaustive. 

It is currently in the early stages of discussion among regulators and other experts how this large source of RWD could provide reliable real-world evidence (RWE) to possibly support regulatory decision making in the field of AIT in the future. 

A recent review by Vogelberg et al. [44] summarizes the current status of RWE for the long-term effect of AIT. It describes a literature search for retrospective cohort assessments of prescription databases in Europe to provide an overview of the methodology, long-term effectiveness outcomes, and adherence to AIT. It is stressed that it will probably take some more years until RWD may be supportive in granting MA for AIT products. 

At this stage, RWE plays an increasing role in regulatory decisions for products for which randomized controlled trials are deemed unethical or unfeasible, such as medicines for very rare diseases or for “precision medicines” [45]. As it is argued that RWD will increasingly be used to not only provide post-authorization safety evidence in rare diseases, but also in a broader use of supporting and contextualizing clinical trial data, many efforts are of importance and will support the advancement of medicines development [45, 46, 47]. 

Many activities are ongoing on EU level, largely steered by the HMA-EMA Joint Big Data Task Force (BDTF) and the HMA-EMA Joint Big Data Steering Group (BDSG). For example, an EU framework for data quality and representativeness is currently being developed as a critical element for realizing the full potential of (big) data and driving regulatory decisions on an EU level [48]. 

With the exception of products for insect venom immunotherapy, confirmatory trials on specific immunotherapy should be performed in a randomized placebo-controlled double-blinded design [11]. In particular, but not limited to, the large placebo effect observed in AIT trials [49], might be a major hurdle in discussion of acceptance of RWE in regulatory decision making for AIT products. 

## Independent official batch release: Benefits and challenges 

The PEI is part of the network of Official Medicines Control Laboratories (OMCLs) that jointly conducts experimental testing of medicinal products in Europe. The European Directorate for the Quality of Medicines & HealthCare (EDQM), a directorate of the Council of Europe, coordinates this network of OMCLs. 

In Germany, the official batch release testing of allergen preparations is regulated according to Section 32 of the German AMG [3]. Responsible for conducting the experimental testing is the PEI. In the past, a large proportion of allergen preparations were not marketed as finished medicinal products but as named patient products (NPPs) which are not subject to official batch testing. In 2008, by Section 1 of the TAO [19], the regulations for finished medicinal products were extended to therapy allergens containing allergens from sweet grasses (without maize), birch, alder, hazel, house dust mites, bee venom, and wasp venom, which – although for individual patients on the basis of a prescription – are produced industrially from prefabricated bulk materials. Section 2 of this regulation subjects these products to batch release testing. 

Requirements for the quality of medicinal products and quality control procedures are defined via the Ph. Eur. monographs. These monographs are regularly adapted to the current state of scientific knowledge, are published in the currently 39 signatory European states [50], and are then legally binding. 

For official batch release, the quality-related, batch-specific documents and test results submitted by the manufacturer are assessed, and samples of the product batches are experimentally tested to determine whether they comply with the specifications of the monograph and in batch-to-batch consistency schemes [9, 51, 52, 53, 54, 55]. Methods described in the pharmacopoeia used in official batch testing investigate product concentration, product identification, allergenic activity, excipients, critical ingredients such as human albumin, or other preservatives. 

A very high product quality is guaranteed on the part of manufacturers through dedicated measures of good manufacturing practice (GMP). Official batch testing is particularly important for materials of biological origin, as natural variations in quality require comprehensive controls. Deviations, e.g., in product stability over a certain storage time, have required batch recalls. Official experimental batch testing and batch release ensures consistent proper quality of AIT products in the German market. 

The Ph. Eur. monograph “Allergen products” requires tests to be performed as late as possible in the manufacturing process [8]. Correspondingly, batch control of AIT products is done on the level of the manufactured bulk for products subject to the TAO, while in authorized preparations it is carried out on the finished product wherever possible. Chemically modified and/or adjuvanted allergens are challenging to access in the finished product for quality control of identity and allergenic activity, because after chemical modification native epitopes are destroyed for immunological detection. Consequently, at this point they are tested at an earlier stage in manufacturing. To study and apply new technologies in the control of medicinal products is feasible and is encouraged [56]. 

Another challenge is that only a few international standards are available that standardize quality controls of allergen products [39, 40, 42]. Each manufacturer has established their own standards (IHRPs) and tests manufactured batches against these. The first recombinant reference standards, Bet v 1 and Phl p 5 and corresponding allergen-specific ELISAs [39, 40] which are established in the Ph. Eur., will now be implemented in the independent official batch release at PEI. After meticulous validation – similar to the approach of BSP090 [39, 40] – further recombinant reference standards and corresponding immunological methods may become available in the next years. 

The ongoing revision of the Ph. Eur. monograph “Allergen products” [8] and the modernization of analytical procedures and alignment with current scientific developments is challenging, as long development and testing phases are preceding. It is important to find a balance between the possibilities of new technologies and the actual need for data for quality assessment while also taking into account the industrial conditions. 

## Harmonization at the EU level 

As has become evident from the previous deliberations, the wide range of aspects that need consideration with respect to AIT medicinal products results in various regulatory and legal requirements to be considered before an AIT product may be placed on the market in any of the EU member states. The most critical step in the regulatory process for allergen medicinal products is the manufacturer’s compilation of data in the dossier for a MA application which is submitted to the national competent authority or EMA and the content of which determines, whether a product can be approved by the competent authority. This application must include detailed information about the product, including its composition, manufacturing process, non-clinical data, clinical trial data, and proposed labeling and packaging. The agencies will then evaluate the application to ensure that it meets all of the necessary requirements to ensure the quality, safety, and efficacy as required for a medicinal product [2]. Yet, as the actual application of these regulations (including Article 5 of Directive 2001/83/EC) varies across the EU, so does the availability of the respective AIT products. Several issues have surfaced in recent years, including divergent views on the acceptance of specific regulatory pathways to be applied and, in relation to this, discrepancies in the type of information and data considered necessary for market approval of AIT products. 

The Co-ordination Group for Mutual Recognition and Decentralised Procedures – Human (CMDh) is responsible for the examination of questions related to the MA of a medicinal product in two or more member states in the EU. To support the harmonization of regulatory procedures for allergen products and thereby enhance the availability of high-quality products in all member states, the CMDh has developed the CMDh Guideline *Recommendation on common regulatory approaches for allergen products* (CMDh/399/2019) [57]. This guideline provides information on the applicable legal basis for allowing any AIT product on the market. This will regularly be a full MA, were a full product-specific data set needs to be provided including data from clinical trials. However, also mixed marketing applications and well-established use applications may be applicable under certain conditions, in which clinical data then need only be provided to a limited extent or can even be substituted by appropriate scientific literature. Furthermore, the use of NPPs is discussed in the guideline, and a framework is defined for which allergens and in which situations the acceptability and use of such NPPs is considered reasonable. Generally, it can be concluded that, for common allergies where a sufficient number of patients should be available for the conduct of clinical trials to verify safety and efficacy of the individual product, a full application is considered necessary. The guideline specifically lists allergen sources where this is considered applicable. Furthermore, the availability of already existing products on the respective markets is taken into consideration. As such, the CMDh states that the preparation and use of NPPs should only be considered in exceptional cases when no alternative authorized allergen products for the treatment of the same allergy are available on the EU market (e.g., where an authorized product for AIT in birch pollen allergy is available, an alternative NPP for birch pollen allergy should not be used). The guideline additionally provides recommendations on the implementation time frame foreseen for the member states to apply the stated recommendations. Here, it is recommended that policies that clarify procedures and transition periods should be developed by the member states within 3 years. According to the guideline and after respective procedures have been developed, applicable transition periods should not exceed a timeframe of 8 years. It is currently unclear to which extent the COVID-19 pandemic has impacted or potentially slowed down respective developments in the member states. 

While these activities by regulators address critical aspects to support harmonization of allergen product regulation across Europe, it has also become evident in recent years that current expectations on the data to be provided in MA procedures may hamper access of products on the market where the availability of sufficient patients to conduct state-of-the-art clinical trials may not be feasible although requested by currently relevant guidelines. As a result, the EMA’s Committee on Medicinal Products in Humans (CHMP), being responsible for elaborating EMA’s opinions on all issues regarding medicinal products for human use, asked the Rheumatology and Immunology Working Party (RIWP) to develop specific guidance on development strategies for allergen products intended for allergies in moderate to low-sized study populations. In a first step, a concept paper was drafted and made available for public consultation in 2019 [58]. Based on this concept paper, the guideline currently in development will include general aspects on allergen product development (patient selection, assessment of efficacy, design of therapeutic studies, safety aspects, and quality considerations) for allergen products where only a limited number of patients is available for development. A public consultation will follow once the guideline has been discussed within RIWP and CHMP. 

## Conclusion 

The field of AIT is shaped by a multitude of important topics and challenges. While this review had to be limited to a selected number of topics from a regulator’s perspective, the entire spectrum of the field is under continuous and intense discussion by many stakeholders, most recently at the 16th triennial International Paul-Ehrlich-Seminar (IPES) (www.pei.de/ipes2023). Exchange platforms like the IPES are of prime importance, as regulatory aspects of AIT are still a work in progress to achieve harmonization among EU member states, but the implementation of the regulatory guidelines is the responsibility of the member states. Also, science and regulation need to be in close exchange in order to stimulate joint further advancement in the development and licensing of AIT products. This is especially the case for new indications, such as asthma prevention and treatment of food allergies using a novel class of active ingredients. 

## Disclaimer 

The views expressed in this review are the personal views of the authors and may not be understood or quoted as being made on behalf of or reflecting the position of the respective national competent authority, the European Medicines Agency, or one of its committees or working parties. 

## Funding 

None. 

## Conflict of interest 

D. Bartel, A. Bonertz, D. Hartenstein, S. Kaul, I. Lauer, V. Mahler, C. Reeb, K. Rösner-Friese, K. Sliva, and J. Zimmer declare no conflict of interest in relation to this work. S. Vieths declares having no direct or indirect interest in pharmaceutical industry/medicinal products. He reports personal fees for published books Schattauer “Allergologie Handbuch”, Elsevier “Nahrungsmittelallergien und Intoleranzen”, Karger “Food Allergy: Molecular Basis and Clinical Practice”; and as Associate Editor of the Journal of Allergy and Clinical Immunology, all outside the submitted work.
